# The knowledge mapping of HIV/AIDS in Indonesians living on six major islands using the Indonesian version of the HIV-KQ-18 instrument

**DOI:** 10.1371/journal.pone.0293876

**Published:** 2023-11-10

**Authors:** Bustanul Arifin, M. Rifqi Rokhman, Zulkarnain Zulkarnain, Dyah Aryani Perwitasari, Marianti Mangau, Saidah Rauf, Rasuane Noor, Retna Siwi Padmawati, Muhammad Nasrum Massi, Jurjen van der Schans, Maarten J. Postma

**Affiliations:** 1 Faculty of Pharmacy, Universitas Hasanuddin, Makassar, South Sulawesi, Indonesia; 2 Department of Health Sciences, University Medical Center Groningen, University of Groningen, Groningen, The Netherlands; 3 Department of Health Behavior, Environment, and Social Medicine, Faculty of Medicine, Public Health and Nursing, Universitas Gadjah Mada, Yogyakarta, Indonesia; 4 Center of Health Behavior and Promotion, Faculty of Medicine, Public Health and Nursing, Universitas Gadjah Mada, Yogyakarta, Indonesia; 5 Institute of Science in Healthy Ageing & healthcaRE (SHARE), University Medical Center Groningen, University of Groningen, Groningen, The Netherlands; 6 Faculty of Pharmacy, Universitas Gadjah Mada, Yogyakarta, Indonesia; 7 Faculty of Medicine, Universitas Syiah Kuala, Banda Aceh, Indonesia; 8 Faculty of Pharmacy, Universitas Ahmad Dahlan, Yogyakarta, Indonesia; 9 Politeknik Kesehatan Kemenkes Maluku, Ambon, Indonesia; 10 Universitas Muhammadiyah Metro, Lampung, Indonesia; 11 Department of Microbiology, Faculty of Medicine, Universitas Hasanuddin, Makassar, South Sulawesi, Indonesia; 12 Department of Economics, Econometrics, and Finance, Faculty of Economics & Business, University of Groningen, Groningen, The Netherlands; 13 Unit of PharmacoTherapy, Epidemiology, and Economics (PTE2), Department of Pharmacy, University of Groningen, Groningen, The Netherlands; 14 Department of Pharmacology and Therapy, Faculty of Medicine, Universitas Airlangga, Surabaya, Indonesia; 15 Center of Excellence in Higher Education for Pharmaceutical Care Innovation, Universitas Padjadjaran, Bandung, Indonesia; Torrens University Australia, AUSTRALIA

## Abstract

Indonesia’s total number of HIV/AIDS cases is still high. Inadequate knowledge about the risk of HIV infection will influence HIV prevention and therapy. This study aimed to map the level of HIV-related knowledge among Indonesians living on six major islands in Indonesia and investigate the relationship between socio-demographic characteristics and HIV/AIDS knowledge. This cross-sectional study used the Bahasa Indonesia version of the HIV Knowledge Questionnaire-18 items (HIV-KQ-18) Instrument. Data collection was done online through the Google form application. A total of 5,364 participants were recruited. The participants from Java had the highest degree of HIV/AIDS knowledge, which was 12.5% higher than participants from Sumatra, Kalimantan, Sulawesi, Papua, and Maluku. Linear regression showed that region, educational level, monthly expenditure, occupation, background in health sciences, and workshop attendance were significantly correlated with HIV knowledge. Participants typically understand that "HIV/AIDS transmission" only happens when sex partners are changed. Additionally, the government still needs improvement in HIV/AIDS education, particularly in the HIV incubation period, HIV transmission from pregnant women to the fetus, and condom use as one method of protection. There are disparities in HIV/AIDS knowledge levels among the major islands of Indonesia. Based on these findings, the government’s health promotion program to increase public awareness of HIV/AIDS must be implemented vigorously. Additionally, in line with our research findings, it is essential to broaden the scope of HIV/AIDS education and promotion materials.

## Introduction

The Joint United Nations Programme on HIV/AIDS (UNAIDS) estimated that in 2022 there were 39.0 million people infected with HIV worldwide, and 37.5 million of them were over 15 years of age [1]. Furthermore, 81% of all people diagnosed with HIV knew their HIV status and 67% were receiving antiretroviral therapy (ART) [2]. In Indonesia, in the last 12 years (data for 2010–2022, for those aged over 15 years), projections of new infections have shown a positive trend, namely from 56,187 new cases to25,740. However, this positive condition is inversely proportional to reports of death rates, which are reported to increase every year. Deaths due to HIV/AIDS in 2010 were 11,971, increasing to 26,501 in 2022 [3]. Numerous studies have reported a variety of HIV/AIDS-related impediments, such as a lack of HIV clinics, stigma and discrimination, and a lack of HIV-related perceptions or knowledge [4–6]. In addition, the level of knowledge about HIV/AIDS can also be another factor that causes undiagnosed HIV as well as increases in the number of new HIV infections [7, 8].

The UNAIDS estimates that two out of every seven new HIV infections worldwide occur among 15–24-year-old [9]. This study also highlights that a contributing factor is the poor degree of knowledge about HIV/AIDS. For example, only one-third of this age group have accurate knowledge of HIV prevention [9], and even some of them think that the disease can be transmitted through mosquito bites or sharing food with someone infected [10, 11]. At the end of 2020, the Ministry of Health of the Republic of Indonesia reported that that 7 out of 10 people with HIV/AIDS are young people in their productive ages of 15–49 [12, 13]. It is important that HIV/AIDS education in Indonesia is continually being improved, especially for the young Indonesians in the productive age range.

Indonesia is an archipelago country with six big islands (Sumatera, Kalimantan, Java, Sulawesi, Bali & Nusa Tenggara, and Maluku & Papua) and hundreds of small islands. According to the findings of studies on health services in Indonesia, there are disparities in terms of topography, demography, and geography characteristics. The regions Bali & Nusa Tenggara and Maluku & Papua were respectively combined because of the geographical location of these islands, which are very close to each other [14, 15]. The influence of socio-demographic inequality on HIV knowledge has been reported to be significant [16]. Due to cultural, socioeconomic, and demographic factors, HIV information dissemination are hindered in Eastern Indonesia, which is located far from the country’s capital city, as is the case in the Middle and Western regions [17]. Understanding the level of HIV/AIDS knowledge by region can be a scientific basis for policymakers in determining target regions for HIV/AIDS education, and ultimately to better control the number of HIV/AIDS cases. Measuring the level of Indonesian knowledge requires measuring tools that are valid, reliable, and have been used globally. Currently, the HIV Knowledge Questionnaire-18 items (HIV KQ-18) is an instrument that has been used to measure the level of HIV/AIDS knowledge of a person or community [18, 19]. This instrument is stable, internally consistent, sensitive to change resulting from intervention, and suitable for the low-literacy community [20, 21]. The HIV-KQ-18 instrument has been validated in several languages, including Indonesian [20], Arabic [22], South Africa [23], and Portugal [24].

This instrument has also been used in several countries, targeting students and certain key populations. Two studies in Southern Ethiopia [25] and Malaysia [19] reported that the majority of male and female students indicated that their knowledge about HIV varied. Men and women have a good understanding of some aspects of HIV transmission but also have certain misconceptions, especially in the context of sexual transmission. Even though there are differences in several questions, statistical tests do not show significant differences between men’s and women’s HIV knowledge [25]. Meanwhile, research with the student population in Malaysia shows that the majority of students (64%) have adequate knowledge about HIV. Factors influencing HIV knowledge include age, gender, and faculty. Findings highlight significant differences in HIV knowledge based on gender and suggest the need for age- and gender-specific educational interventions to address misconceptions about HIV, how it is transmitted, and prevention [19]. Meanwhile, research using instruments targeting key populations was conducted in Malaysia and Nigeria. In Indonesia itself, research using the HIV-KQ-18 instrument was actually carried out and published in 2019. In this study in Lampung, Indonesia, it was reported that there was a significant relationship between the HIV stigma variable, HIV knowledge, and the HIV risk behaviour variable. The most dominant factor influencing motivation to test for HIV concerned risk behaviour, location of the women who live with HIV and engagement in risky behaviour [26].

The first objective of this study was to map the level of HIV-related knowledge among Indonesians who live on the six major islands in Indonesia, using the Bahasa Indonesian version of the HIV-KQ-18 instrument [20]. Secondly, this study also aimed to investigate the relationship between various determinants, such as socio-demographic characteristics, and HIV/AIDS knowledge. We expect that the results of this study will serve as a scientific source for the government to use in formulating HIV/AIDS education policies for specific populations, thereby optimising and targeting government budget allocations.

## Methods

### Participants

Although 15–24 years of age is a high-risk range for HIV/AIDS exposure, we only recruited participants with minimum age of 17 years due to ethical considerations. This is owing to the fact that some items require a more comprehensive explanation to prevent participants under the age of 17 from misunderstanding the information. We limited the data collection date range for this research to 12 months, from September 2020 to August 2021.

In this study, to expand the number of persons who might participate, we optimized the acquaintances/colleagues/relations of each Indonesian author in this study to assure the representation of each island that served as the locations of data gathering. MRR, ZZ, DAP, RN, and RSP are Western Indonesian authors (Java and Sumatra). BA, MM, and MNM are authors from Central Indonesia (Kalimantan, Sulawesi, Bali, and Nusa Tenggara), while SR is an author from Eastern Indonesia (Maluku and Papua). Additionally, it was the responsibility of each author in each region to guarantee that at least one rural and one urban location were represented among participants that completed our instrument link.

According to Daniel’s formula for calculating sample sizes for cross-sectional studies, the minimum total sample for each study location was estimated at 385 participants [27] The minimum sample size was calculated using the following parameters: 95% level of confidence, 1.96 for the Z1-α/2 value, 0.5 expected proportion, and 0.05 absolute error or precision.

Prior to participants completing the study’s questionnaires, these assessments began with a general explanation of the project’s goals and a request for signed informed consent to expressed willingness to participate in this study. This research has been approved by the Ethics Committee ‘*Komite Etik Penelitian*’ (KEP) Universitas Ahmad Dahlan, Yogyakarta, Indonesia (012007028, 22 September 2020 amendment 26 November 2021).

### Instrument

The HIV-KQ-18 instrument consists of 18 question items about HIV transmission and prevention. For each correct answer, a score of 1 was assigned (maximum 18/18), while for incorrect answers a score of 0 was counted. For participants who answered ’don’t know, a score of 0 was also counted. This instrument has been validated into Bahasa Indonesia and has passed the "cross cultural adaption" test [20]. Notably, Prof. Michael P. Carey, Ph.D., has given his approval for the license to use this instrument (via email on February 11, 2020).

### Procedure

This cross-sectional study utilized the Bahasa Indonesia version of the HIV Knowledge Questionnaire-18 (HIV-KQ-18) Instrument [20]. Multiple social media platforms, including WhatsApp, Facebook messenger, email, Instagram, and Twitter, were utilized to collect data online using the Google Form application. We removed multiple entries for participants based on their initials and date of birth if they had joined through multiple channels.

All of the Indonesian researchers distributed the online Google form link to all of their colleagues, who then gave it to their students when they taught in class. The students were asked to distribute it to their family and colleagues. Before the statement of willingness to participate, participants were explained the research objectives in detail and their understanding was checked. Furthermore, they provided socio-demographic data, including initials, age, occupation, monthly expenses, education, experience with participating in HIV/AIDS seminars, health educational background, and marital status. We also provided opportunities for participants to express their opinions about HIV/AIDS in Indonesia and asked which questions they think were the most difficult? Based on this question, we determined which items were perceived as the most difficult items by participants. In order to increase the reach of this link’s distribution, we also informed several other colleagues from various departments.” In order to increase the reach of this link’s distribution, we also informed several other colleagues from various departments.

### Statistical analysis

Data were analyzed descriptively. Participants’ knowledge of HIV/AIDS was reported using mean and standard deviation (SD). The differences in participants’ knowledge based on their region and different socio-demographic characteristics were analyzed by using an independent t-test or one-way ANOVA. If one-way ANOVA indicated a significant difference, a Bonferroni post hoc test was also done. Multiple linear regression was used to investigate the predictive variables associated with participants’ knowledge of HIV/AIDS. Descriptive statistics were employed in the data analysis and the statistical analysis was performed using SPSS version 26 (IBM Corp., Armonk NY, USA). All data were reported as means with SD, with *p*<0.05 denoting statistical significance.

## Results

### Participant characteristics

In total, there were 5,364 participants in this study. Of the participants, 60% were between the ages of 17 and 25. This tendency may be noticed not only on two islands with over 1,000 participants but also on the islands of ’Bali and Nusa Tenggara,’ where the number of participants was over 7 times lower than on Sulawesi. Furthermore, when looking at the types of involvement by gender, each region always had more female participants than males (*p* < .001). Approximately 3,000 participants stated that they had not worked, as evidenced by the percentage with a bachelor’s degree, which was also greater than other educational levels, and almost 43% of participants said their average monthly expenditure was less than 2 million rupiahs (USD 143). This participant group, it is assumed, consists of persons without a steady job. During data collection, we also asked about the participants’ health education backgrounds. Although the proportions of the two groups, i.e. participants’ with and without health education backgrounds were nearly identical in general, only 23% of the 564 Sumatran participants claimed that they had studied in the health sector (doctor, pharmacist, dentist, nurse, or public health graduate). Details of these socio-demographic characteristics are presented in Table 1.

**Table 1:** Characteristics of participants from 6 regions in Indonesia.

Variables	Total N (%)	%					
		Java (n = 1,209)	Sumatera (n = 791)	Kalimantan (n = 399)	Bali and Nusa Tenggara (n = 370)	Sulawesi (n = 1,735)	Maluku and Papua (n = 860)
Overall	5,364 (100)	22.5	14.7	7.4	6.9	32.3	16.0
Age, n = 5,362							
17–25 years	3,104 (57.9)	44.8	48.5	80.5	75.4	56.8	68.8
25–35 years	1,359 (25.3)	34.0	28.7	9.3	16.2	26.5	19.1
35–45 years	652 (12.2)	14.6	16.1	4.5	6.5	13.5	8.5
>45 years	247 (4.6)	6.6	6.4	5.8	1.9	3.2	3.6
Gender							
Female	3,486 (65.0)	60.0	64.6	70.7	72.7	61.3	74.0
Male	1,878 (35.0)	40.0	35.4	29.3	27.3	38.7	26.0
Education level							
Up to senior high school	1,247 (23.2)	18.8	20.5	29.6	20.0	24.7	27.7
Bachelor’s degree	3,557 (66.3)	60.9	64.7	59.4	71.1	70.4	68.4
Postgraduate	560 (10.4)	20.3	14.8	11.0	8.9	5.0	4.0
Marital status, n = 3,480							
No	2,318 (43.2)	46.5	20.4	39.8	71.1	35.3	65.2
Yes	1,162 (21.7)	31.7	22.1	9.3	8.9	21.4	19.0
Monthly expense (Rupiah), n = 3,508							
< 2 million	2,291 (42.7)	38.0	21.5	33.6	61.9	41.2	67.9
2–3 million	528 (9.8)	15.6	7.8	5.3	10.5	8.6	7.9
3–4 million	245 (4.6)	6.9	5.2	4.5	3.5	3.1	4.2
4–5 million	168 (3.1)	5.8	4.6	2.3	2.7	1.6	1.9
>5 million	276 (5.1)	12.2	3.5	3.5	3.0	3.1	2.4
Occupation							
Not working	2,916 (54.4)	41.8	48.8	76.4	69.7	51.4	66.3
Entrepreneur	1,128 (21.0)	35.7	23.3	9.3	17.6	17.3	12.7
Permanent staff	1,320 (24.6)	22.5	27.9	14.3	12.7	31.2	21.0
Background in health sciences							
No	2,649 (49.4)	49.6	71.3	47.4	35.9	47.7	39.0
Yes	2,715 (50.6)	50.4	28.7	52.6	64.1	52.3	61.0
Workshop, n = 5,363							
No	3,588 (66.9)	70.2	70.2	73.7	55.7	64.6	65.6
Yes	1,775 (33.1)	29.8	29.8	26.3	44.1	35.4	34.4

### The distribution of correct to each HIV-KQ-18 item

**Fig 1:**
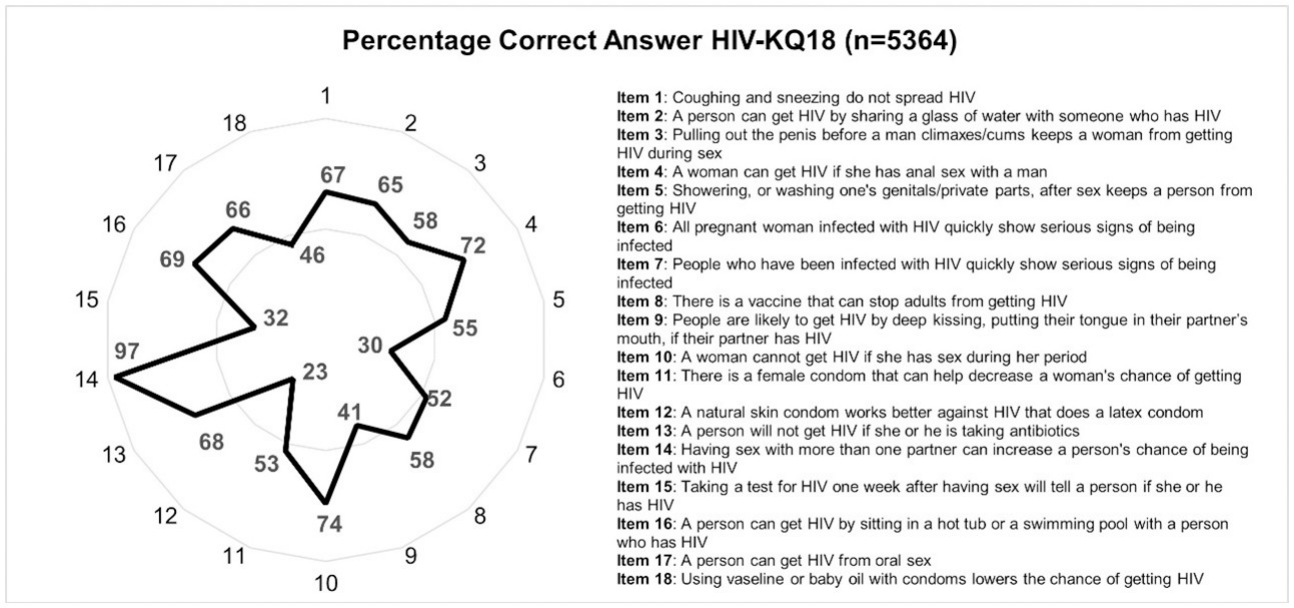
The distribution of answers of each HIV-KQ-18 item from 6 regions in Indonesia. *Notes*. Item 1: Coughing and sneezing do not spread HIV; Item 2: A person can get HIV by sharing a glass of water with someone who has HIV; Item 3: Pulling out the penis before a man climaxes/cums keeps a woman from getting HIV during sex; Item 4: A woman can get HIV if she has anal sex with a man; Item 5: Showering, or washing one’s genitals/private parts, after sex keeps a person from getting HIV; Item 6: All pregnant woman infected with HIV quickly show serious signs of being infected; Item 7: People who have been infected with HIV quickly show serious signs of being infected; Item 8: There is a vaccine that can stop adults from getting HIV; Item 9: People are likely to get HIV by deep kissing, putting their tongue in their partner’s mouth, if their partner has HIV; Item 10: A woman cannot get HIV if she has sex during her period; Item 11: There is a female condom that can help decrease a woman’s chance of getting HIV; Item 12: A natural skin condom works better against HIV that does a latex condom; Item 13: A person will not get HIV if she or he is taking antibiotics; Item 14: Having sex with more than one partner can increase a person’s chance of being infected with HIV; Item 15: Taking a test for HIV one week after having sex will tell a person if she or he has HIV; Item 16: A person can get HIV by sitting in a hot tub or a swimming pool with a person who has HIV; Item 17: A person can get HIV from oral sex; Item 18: Using vaseline or baby oil with condoms lowers the chance of getting HIV.

Fig 1 shows that the distribution of answers was generally consistent across sites. Item number 14 (having sex with more than one partner can increase a person’s change of being infected with HIV) was answered correctly by nearly all of the participants. This finding supports our initial hypothesis that most participants will correctly respond to item number 14 in this survey. The majority of participants (almost 100%) were aware that "unfaithfulness to one’s marriage spouse" or "often changing sex partners" can raise one’s risk of contracting HIV/AIDS. Meanwhile, numerous participants continued to give incorrect answers to items on condoms, the transmission of HIV/AIDS from infected pregnant women to the fetus, and the length of the HIV/AIDS incubation period (items 6, 12 and 15).

### The participant’s level of HIV knowledge based on regions

Table 2 demonstrates the participants’ level of HIV/AIDS knowledge and its association with their regions and sociodemographic characteristics. Of all of the participants, those from Java had the highest degree of HIV/AIDS knowledge, which was significantly different from Sumatra, Kalimantan, Sulawesi, Maluku, and Papua (*p*<0.01), but not from ’Bali and Nusa Tenggara’. In general, all sociodemographic characteristics were related to knowledge level, except for gender.

**Table 2:** The distribution of knowledge based on regions and participants’ characteristics.

Variables	Total Mean (SD)	HIV-KQ-18 score, Mean (SD)					
		Java	Sumatera	Kalimantan	Bali and Nusa Tenggara	Sulawesi	Maluku and Papua
Overall	10.3 (4.0)	11.2 (3.8) ^a^	9.9 (4.2)**	10.3 (4.1)**	10.7 (4.0)	9.8 (4.0)**	9.9 (4.0)**
Age							
17–25 years	9.7 (3.8) ^a^	10.5 (3.7) ^a^	9.0 (3.8) ^a^	9.8 (4.2) ^a^	9.9 (3.8) ^a^	9.6 (3.8) ^a^	9.4 (3.9) ^a^
>25–35 years	11.2 (4.0)**	11.8 (3.7)**	10.9 (4.2)**	10.9 (3.5)	12.8 (3.4)**	10.4 (3.9)**	11.5 (4.2)**
>35–45 years	11.0 (4.3)**	11.9 (3.6)**	11.2 (4.5)**	13.4 (2.3)**	14.6 (3.0)**	9.6 (4.4)	10.8 (3.9)**
>45 years	10.3 (4.4)	11.1 (4.0)	10.1 (5.0)	13.3 (2.5)**	13.3 (1.7)*	8.5 (4.7)	9.2 (3.9)
Gender							
Female	10.2 (3.9)	11.2 (3.6)	9.9 (4.2)	10.0 (4.1)	10.4 (3.9)*	9.8 (3.9)	10.0 (4.0)
Male	10.3 (4.1)	11.1 (4.0)	10.0 (4.2)	10.9 (4.1)	11.5 (4.0)	9.8 (4.1)	9.7 (4.1)
Education level							
Up to senior high school	9.1 (4.1) ^a^	9.1 (4.0) ^a^	8.9 (4.2) ^a^	9.8 (4.1) ^a^	10.0 (4.5) ^a^	8.6 (4.0) ^a^	9.3 (4.0) ^a^
Bachelor’s degree	10.3 (3.9)**	11.3 (3.6)**	9.9 (4.2)**	10.1 (4.2)	10.5 (3.7)	10.1 (3.9)**	10.1 (4.0)*
Postgraduate	12.4 (3.4)**	12.9 (3.0)**	11.6 (3.9)**	13.0 (2.7)**	13.8 (3.5)**	11.7 (3.5)**	12.0 (3.4)**
Marital status							
No	10.1 (3.9)**	10.7 (3.6)*	9.5 (4.0)**	10.5 (4.0)**	10.3 (3.9)**	10.0 (3.9)	9.6 (3.9)**
Yes	10.9 (4.3)	11.4 (4.0)	11.8 (4.3)	12.2 (2.9)	12.7 (3.8)	9.9 (4.5)	10.6 (4.4)
Monthly expense (in Rupiah)							
<2 million	9.7 (3.9) ^a^	9.8 (3.7) ^a^	10.3 (4.1) ^a^	10.3 (4.0) ^a^	9.8 (3.8) ^a^	9.6 (4.0) ^a^	9.5 (3.9) ^a^
2–3 million	11.0 (4.0)**	11.4 (3.4)**	10.1 (4.5)	12.0 (3.2)	12.5 (3.7)**	10.5 (4.4)*	11.1 (4.6)**
3–4 million	11.3 (4.2)**	12.2 (4.1)**	11.6 (4.3)	10.9 (4.2)	11.5 (4.2)	10.4 (3.7)	10.3 (4.9)
4–5 million	11.9 (3.7)**	12.6 (3.3)**	11.0 (4.0)	11.8 (2.9)	12.1 (5.3)	11.8 (4.1)**	11.2 (3.4)
>5 million	12.6 (3.7)**	12.5 (3.4)**	12.8 (4.3)**	13.5 (1.7)**	12.1 (4.1)	12.2 (4.3)**	13.2 (3.9)**
Occupation							
Not working	9.6 (3.9) ^a^	10.5 (3.6) ^a^	9.1 (3.9) ^a^	9.8 (4.2) ^a^	10.0 (3.8) ^a^	9.4 (3.7) ^a^	9.3 (3.9) ^a^
Entrepreneur	10.5 (4.1)**	11.1 (3.8)*	9.9 (4.5)	10.8 (3.5)	11.6 (4.2)**	9.6 (4.2)	10.5 (4.3)*
Permanent staff	11.5 (3.9)**	12.6 (3.4)**	11.4 (4.1)**	12.6 (3.0)**	13.7 (3.0) **	10.6 (4.1)**	11.5 (3.7)**
Background in health sciences							
No	9.2 (4.1)**	10.2 (3.9)**	9.2 (4.2)**	8.9 (4.2)**	9.6 (4.3)**	8.5 (4.0)**	8.9 (4.4)**
Yes	11.3 (3.6)	12.2 (3.3)	11.8 (3.7)	11.5 (3.6)	11.3 (3.6)	11.0 (3.5)	10.6 (3.6)
Workshop							
No	9.8 (4.1)**	10.8 (3.8)**	9.4 (4.2)**	9.8 (4.1)**	10.4 (3.9)	9.4 (4.1)**	9.5 (4.1)**
Yes	11.1 (3.7)	12.1 (3.4)	11.2 (3.9)	11.6 (3.8)	11.2 (4.1)	10.4 (3.6)	10.9 (3.7)

Note:

^a^ This subgroup used as reference for the post-hoc analysis; SD, standard deviation;

*p<0.05;

**p<0.01

In comparison to other age groups, those aged >25–45 years have a higher level of knowledge of HIV/AIDS. On all islands, this age group’s knowledge score differed significantly from that of the 17–25 years age group, except for Kalimantan (vs. >25–35 years) and Sulawesi (vs. >35–45 years). Remarkably, participants in Kalimantan and ’Bali and Nusa Tenggara’ who were >35 years showed substantially higher HIV/AIDS knowledge scores than those aged 17–25 years (*p*<0.01 and *p*<0.05, respectively).

Overall, participants with a postgraduate degree scored higher than those with only a senior high school or less. The average level of knowledge of HIV/AIDS followed the same pattern on all islands. Also, overall data showed that married participants had significantly better HIV/AIDS knowledge than those who were not married (*p*<0.01), which was found on every island except Sulawesi (*p*>0.05).

The analysis of the knowledge about HIV/AIDS among all participants relating to their monthly expenditure reveals that the higher their expenditure, the more knowledgeable they are. However, there are distinct patterns on each island. Participants in Java showed a considerably higher level of knowledge if monthly expenses >2 million compared to those who spent <2 million rupiahs per month. Meanwhile, Kalimantan and Sumatra have a similar difference that was however only visible for individuals who spend >5 million every month. Interestingly, the group that spends 2–3 million rupiahs per month in "Bali and Nusa Tenggara" has the highest level of knowledge and is significantly different from the group that spends <2 million.

In general, non-working participants possessed the least knowledge of HIV/AIDS compared to entrepreneurs and permanent staff. This was also observed among participants from Java region, ’Bali and Nusa Tenggara’ regions, as well as for Maluku and Papua. However, entrepreneurial participants in Sumatra, Kalimantan, and Sulawesi have a similar degree of knowledge to non-workers.

Participants with a background in health education and a history of attending HIV/AIDS programs scored higher. This is evident both for the general population and for the individual islands. Surprisingly, the level of HIV/AIDS knowledge among participants in ’Bali and Nusa Tenggara’ did not differ significantly between those who attended the workshop and those who did not.

### Participant perceptions of HIV-KQ-18 instrument items

In addition to hoping that HIV/AIDS education will be intensified with a wider reach, there are a few subjects that would like to know more about specific topics in depth: (i) more about condoms is needed, such as the chemicals used in their manufacture, since many participants currently only know that they are made of rubber. Additionally, they want to know in more detail if using condoms with other lubricants, such as baby oil, can improve their performance, which has nothing to do with HIV/AIDS; (ii) Participants typically anticipate that infants born to HIV-positive mothers will undoubtedly carry the virus themselves. In fact, given the advancements in HIV treatment, these infants may still be rescued and cured with the right care; and (iii) Another suggestion is for more information concerning the so-called ‘window period’, since participants do not really understand the incubation period of HIV.

### The predictor variables of HIV knowledge

Table 3 summarizes the results of the multiple linear regression study. Six of ten variables, including region, educational level, monthly expenditure, occupation, background in health sciences, and workshop attendance, were found to be significantly correlated with HIV knowledge. However, this model is only marginally capable of predicting the level of knowledge in all participants (R square = 17.5%).

**Table 3:** Predictors of HIV knowledge.

Variables	Unstandardized Coefficients	Standardized Coefficients	*p*-value
Regions	-0.154	-0.067	<0.001
Age	-0.076	-0.016	0.508
Gender	0.238	0.028	0.089
Education level	0.531	0.073	<0.001
Marital status	-0.160	-0.019	0.436
Monthly expense	0.527	0.162	<0.001
Occupation	0.522	0.108	<0.001
Background in health sciences	2.057	0.253	<0.001
Workshop	1.034	0.120	<0.001
Constant	7.763		<0.001

## Discussion

Our study proved that although there are disparities in knowledge levels amongst islands in Indonesia, the distribution of the knowledge follows a remarkably similar pattern. The uniformity in the proportion of accurate responses across all islands is most likely due to Indonesia’s HIV/AIDS preventive policy being structured and coordinated from the national to local government levels [28]. According to the findings of sociodemographic studies based on multiple linear regression analysis, the following variables influence Indonesians’ knowledge of HIV/AIDS: (i) region/island; (ii) education level; (iii) monthly expenses; (iv) occupation; (v) background in health education; and (vi) participation in seminars or workshops on the topic. Furthermore, this study found that most participants know that HIV can be caused by having sex with more than one partner. The findings corroborate a previous study conducted in Yogyakarta, Indonesia [29]. Although addressing sexuality is widely considered taboo in Indonesia [30, 31], it has been stated that education regarding free sex and HIV began around two decades ago in the country, even at the elementary school level [32].

Remarkably, less than a quarter of participants were familiar with the use and varieties of condoms. This indicates a dearth of condom knowledge in the general population of Indonesia. It is common knowledge that HIV/AIDS prevention and promotion programs are standardized, i.e., based on a plan approved by government ministries [33, 34]. In the Asia-Pacific region, including Indonesia, the HIV epidemic is concentrated among the significant populations of men who have sex with men (MSM), female sex workers, people who inject drugs (PWID), and transgender women (*waria*), making HIV education and treatment a national priority for these populations [35, 36]. Several HIV-related studies undertaken in Indonesia’s key populations have established an association between condom awareness and usage frequency. Greater understanding of condoms is associated with increased prevalence of their use [37–41]. According to the findings of this study, there is an urgent need to educate not only targeted populations but also the general community about condoms, despite the fact that explaining or socializing people about how to use condoms properly remains a challenge.

In addition, we also discovered that only around a third of all participants were able to accurately respond to questions about the HIV virus’s incubation period (window period) and transmission from pregnant woman to the fetus. Notably, Indonesia is not the only nation dealing with this problem, while the lack of public awareness regarding HIV symptoms and mother-to-child transmission routes has been noted in various Asian and African countries, particularly among non-HIV/AIDS populations [42–47]. These findings should be considered when developing HIV/AIDS instructional materials. In summary, we recommend that HIV/AIDS education materials focus on: (i) mother-to-child HIV/AIDS transmission; (ii) condom usage to prevent HIV/AIDS transmission; and (iii) understanding of the incubation period of HIV. Finally, we advise that HIV/AIDS education be targeted at the undergraduate I for those whose highest level of education is Senior High School, including those with backgrounds in disciplines other than health education. Additionally, Eastern Indonesia needs to be included in the geographic area covered by HIV/AIDS education (more specifically, remote and rural areas). Regarding teaching materials, we seriously encourage strengthening awareness on condom use, which not only works to prevent pregnancy but also HIV, particularly among adolescents.

The regression analyses demonstrated that regions, education level, monthly expense, occupation, background in health science, and workshops on HIV/AIDS were determinant factors for HIV knowledge. As shown in Table 2, Javanese and ’Bali and Nusa Tenggara’ participants have a significantly higher average level of knowledge (12%) than participants from Sumatra, Kalimantan, Sulawesi, Papua, and Maluku. Over half of those infected with HIV in Indonesia are found in Java and Bali [48]. Furthermore, the majority of urban concentration areas are located in both regions [49]. It is generally recognized that living in larger cities and having a higher prevalence of HIV provide greater availability and access to sources of HIV/AIDS information, resulting in a better understanding of the disease [50–53].

In line with previous investigations performed on various populations [43, 50, 51, 54–59], the present study found that for overall participants, the higher their degree, the higher their monthly expenses, having permanent jobs, having a health educational background, and having attended HIV/AIDS seminars/ workshop, then the more knowledgeable they are. However, the results of the one-way ANOVA analysis for participants’ monthly expenses and experience in HIV/AIDS workshop per island have shown a difference in the trends of participants from Bali and Nusa. It is essential to recognize that, the R2 value of this study is relatively low. Several studies in countries with a high incidence of HIV highlight additional knowledge-related characteristics that have not been investigated in this study, including HIV awareness and behavior, stigma, social media use, and HIV testing history [43, 46, 60–62]. Thus, it is challenging to develop a complete picture of the factors impacting the HIV knowledge of the general population of Indonesia.

The participants with the highest level of HIV knowledge were the ones who spend between 2–3 million rupiahs per month in Bali and Nusa. Bali’s population is more than 90% economically active and almost evenly distributed across all educational levels [63]. Bali is a target area for national tourism. It was interesting to note that tourism is linked to a rise in knowledge on reproductive health issues including HIV/AIDS and sexually transmitted illnesses [64–66]. Recognizing the serious threat, the Bali community and government have developed a comprehensive HIV/AIDS promotion and prevention program that involves community empowerment at all levels [67–69].

To date, we have yet to locate a comparable study in Indonesia, particularly with the number of participants representing nearly all regions of Indonesia (West, Central, and East). We compared our results in the 17–25 age range (n = 3,104) with a study of university students in Malaysia (n = 405) [19]. The mean score of HIV-AIDS knowledge among Indonesian students was 10.8, with Java (n = 1,209) having the highest score (11.2/18) and Sulawesi (n = 1,735) having the lowest (9.8/18). One study in Jakarta, Indonesia, conducted in 2019 used the HIV-KQ-18 instrument to assess participants’ knowledge of HIV/AIDS. There were 81 participants in total, with an equal number of men and women. According to the study’s findings, 56% of participants had low test scores, with a mean of 8.20 out of 18. There were 19 participants in the 18–25 age range, and 10 of them scored in the low range [70]. Comparing these results to a study with a student population in Malaysia revealed that their mean HIV/AIDS knowledge score was 7/18 [19]. In addition, we did not detect a significant difference between males and females, although the Malaysian study found that male students had greater knowledge than female students [19]. In addition, an analysis of the level of HIV/AIDS awareness among university students in South-eastern Ethiopia (n = 442.67% male) revealed that more than half of the students showed a low level of HIV/AIDS knowledge. Field of study, year of study (duration of study), and monthly income were three significant factors. In that study, it is advised that HIV/AIDS teaching at universities should involve several years of study and that risk reduction measures should prioritize behavior modifications [44].

Another finding that is also interesting to discuss is the item that is most easily answered correctly by the participants. Our study found that almost 100% of Indonesian participants agreed that ’Having sex with more than one partner can increase a person’s chance of becoming infected with HIV (item number 14).’ Comparing this finding to a research on HIV Knowledge and Associated Factors among Internet-Using Men Who Have Sex with Men (MSM) in South Africa and the United States revealed that nearly 100 percent of participants answered questions 16 (a person can get HIV by sitting in a hot tub or a swimming pool with a person who has HIV) and 17 (A person can get HIV from oral sex) correctly [70]. In addition to continuing with the ’faithful to 1 married couple’ campaign, the HIV/AIDS education topics that have been implemented in Indonesia should also emphasize the possibility of other transmission routes, such as oral and anal sex, as strongly suggested by this study.

Despite the fact that this study remains limited by online recruitment of participant and the uneven distribution of participants by region, one of the research’s strengths is the location of the data collection in all main Indonesian islands, where each island is represented by at least one urban region (provincial capital) and one rural area (city/ district). Furthermore, we found that Indonesian people continue to hold the belief that only "having several sex partners (free sex) causes and transmits HIV/AIDS." In fact, HIV transmission can actually happen due to a variety of causes, such as from a pregnant woman to the fetus. Also, negative stigma against those who are living with HIV/AIDS is very likely to emerge due to a lack of understanding of this disease. The fact that young adult people and adolescents are more habituated to using condoms as a method of contraception is more known than condoms’ effectiveness in preventing the transmission of HIV. In fact, this age demographic is the most vulnerable compared to others. Beyond that group, the Indonesian authorities still have significant work to do in the area of condom education. A qualitative study in Indonesia on condom use with 42 male participants who had sexual relations with female commercial sex workers (FCSW) confirmed that some of the reasons they eventually decided not to use condoms despite knowing that doing so could increase their risk of contracting HIV/AIDS are as follows: (i) limited sexual pleasure obtained during biological intercourse; (ii) difficulty in accessing and relatively expensive condom prices; (iii) condoms are perceived as having no benefit; (iv) low self-awareness to avoid the negative effects of not using condoms; and (v) feeling embarrassed to purchase condoms [40]. In addition, an analysis of condom use trends among female commercial sex workers in Bali, Indonesia found that FCSWs with higher rates of sexual transactions are more knowledgeable of condom use during risky activities than those with lower/middle rates. Moreover, the role of ’a pimp’ in the transaction also plays an important role [40]. Hence, our results can be used as a scientific resource to inform and generate more pertinent HIV/AIDS condom education.

## Conclusions

We conclude that: (i) there was a strong association between socio-demographic characteristics and knowledge mapping of HIV/AIDS on six major islands of Indonesia; (ii) to achieve success in the health promotion of HIV/AIDS, programs should be designed to increase not only the knowledge domain, but also the socio-demographic characteristics in communities; and (iii) in order to disseminate HIV/AIDS education to the broader community, posters, booklets, and brochures (made in a more attractive and durable form, for example, a plastic fan or a folding fan) must also be distributed to schools, academic institutions, and public communities.

## Supporting information

S1 Dataset (XLSX)
